# Innate Lymphoid Cells in Human Pregnancy

**DOI:** 10.3389/fimmu.2020.551707

**Published:** 2020-11-30

**Authors:** João Mendes, Ana Luísa Areia, Paulo Rodrigues-Santos, Manuel Santos-Rosa, Anabela Mota-Pinto

**Affiliations:** ^1^ Faculty of Medicine, Coimbra Institute for Clinical and Biomedical Research (iCBR), University of Coimbra, Coimbra, Portugal; ^2^ Faculty of Medicine, Center of Investigation in Environment, Genetics and Oncobiology (CIMAGO), University of Coimbra, Coimbra, Portugal; ^3^ Faculty of Medicine, General Pathology Institute, University of Coimbra, Coimbra, Portugal; ^4^ Center for Innovation in Biomedicine and Biotechnology (CIBB), University of Coimbra, Coimbra, Portugal; ^5^ Obstetrics Department, Coimbra University Hospital Center, Coimbra, Portugal; ^6^ Faculty of Medicine (FMUC), Institute of Immunology, University of Coimbra, Coimbra, Portugal; ^7^ Center for Neuroscience and Cell Biology (CNC), Laboratory of Immunology and Oncology, University of Coimbra, Coimbra, Portugal

**Keywords:** innate lymphoid cells, innate immune response, inflammation, pregnancy, preterm birth

## Abstract

Innate lymphoid cells (ILCs) are a new set of cells considered to be a part of the innate immune system. ILCs are classified into five subsets (according to their transcription factors and cytokine profile) as natural killer cells (NK cells), group 1 ILCs, group 2 ILCs, group 3 ILCs, and lymphoid tissue inducers (LTi). Functionally, these cells resemble the T helper population but lack the expression of recombinant genes, which is essential for the formation of T cell receptors. In this work, the authors address the distinction between peripheral and decidual NK cells, highlighting their diversity in ILC biology and its relevance to human pregnancy. ILCs are effector cells that are important in promoting immunity, inflammation, and tissue repair. Recent studies have directed their attention to ILC actions in pregnancy. Dysregulation or expansion of pro-inflammatory ILC populations as well as abnormal tolerogenic responses may directly interfere with pregnancy, ultimately resulting in pregnancy loss or adverse outcomes. In this review, we characterize these cells, considering recent findings and addressing knowledge gaps in perinatal medicine in the context of ILC biology. Moreover, we discuss the relevance of these cells not only to the process of immune tolerance, but also in disease.

## Introduction

From the immunology point of view, we may consider the fetus as a semi-allograft concept initially put forward in 1953 by Sir Peter Medawar (1). Accordingly, taken from the knowledge attained in transplantation science, the trophoblast carrying paternal antigens must invade the maternal uterine mucosa, in a process called implantation, while escaping immune defense mechanisms against the alloantigen. This is the first paradox in the biology of pregnancy (2).

Indeed, there are three major phases in pregnancy, involving the immunologic response: implantation of the trophoblast, widely regarded as an inflammatory process; followed by a protective anti-inflammatory milieu, needed throughout the whole development of the fetus; and finally, labor itself, which is also regarded as an inflammatory event (3–9). During pregnancy, differences in inflammatory states relate to different cytokine profiles. Evidence in the literature supports a shift from a T helper 1 (Th1) cytokine profile to a Th2 profile favoring a humoral response (10). However, other cell types contribute to the immune regulation of pregnancy, such as T regulatory cells, IL-17-producing cells, and tissue resident cells (11, 12).

The majority of scientific investigation has focused on the T cell repertoire and on the balance between Th1, Th2, and Th17 cytokines (13–16); nonetheless, it is evident that the innate component of the immune system has a preponderant role in pregnancy (17, 18). Moreover, the classic Th1/Th2 paradigm fails to explain the immunomodulatory actions of locally secreted cytokines.

In this review, we focus on innate immune responses during pregnancy, considering a recently categorized set of cells called innate lymphoid cells (ILCs).

## Innate Lymphoid Cells

**Figure 1:**
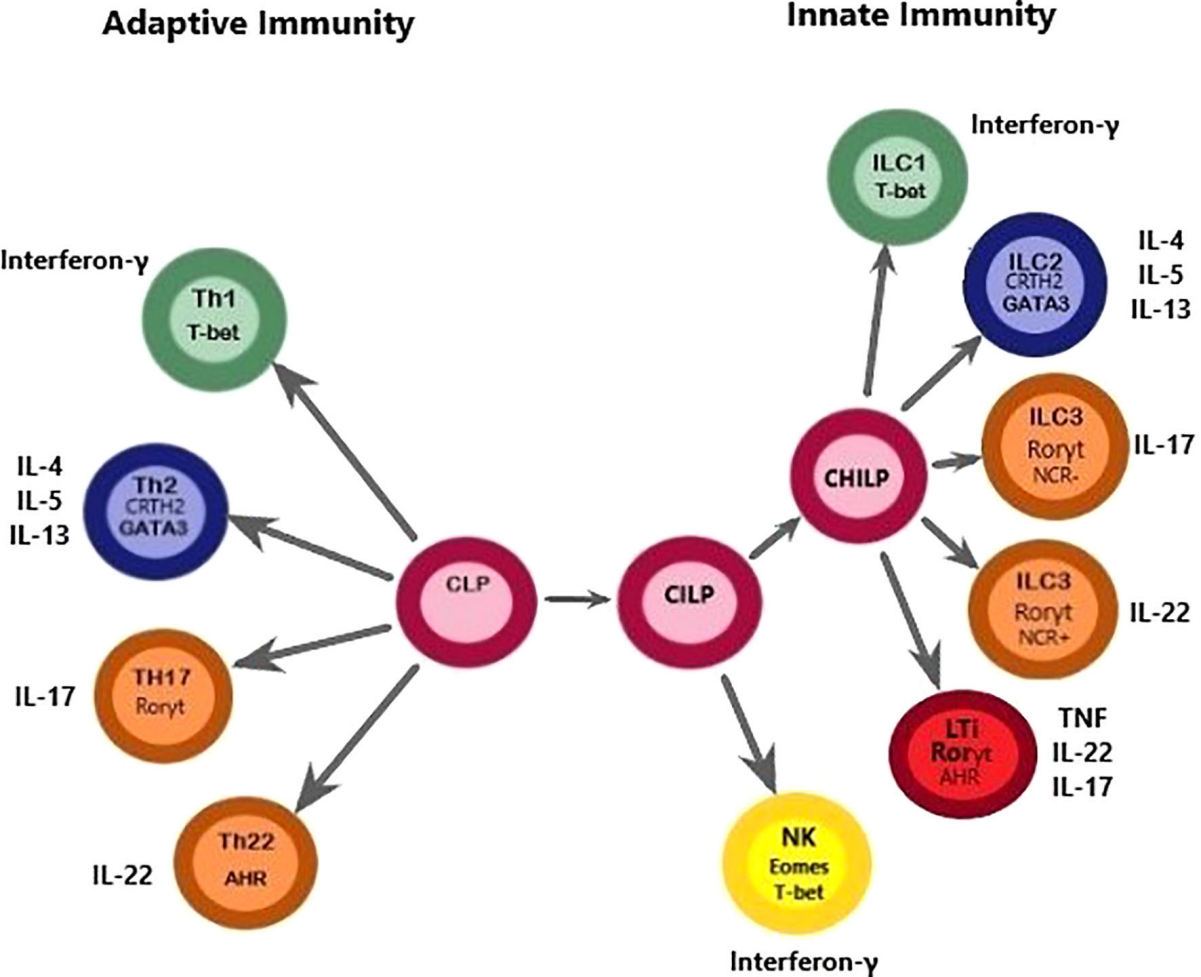
This figure depicts a proposed model for the differentiation paths of ILCs, highlighting the similarities with Th cells regarding common transcription factors. A common lymphoid progenitor (CLP), originated from a hematopoietic stem cell (HSC) can give rise to adaptive and innate lymphocytes. However, it should be noted that this figure was simplified to convey the message that Th cells and ILCs are of lymphoid origin because Th cell populations do not differentiate directly from CLPs. Downstream of the CLP, a common ILC precursor (CILP) then divides into 1) a branch that differentiates into NK cells and 2) a branch that generates a common helper-ILC precursor (CHILP). The CHILP further differentiates toward a different branch of the ILC family, namely ILC1, ILC2, and ILC3, and generates an LTi population. This figure highlights similarities between ILCs and Th cells. The classification of ILCs is based on functional criteria. ILCs functionally resemble adaptive lymphocytes with the distinction that ILCs lack antigen-specific receptors. Instead, ILCs are known to exert their effects through the production of cytokines and cell surface molecules with important consequences for tissue homeostasis, inflammation, and disease. Dysregulation or expansion of pro-inflammatory ILC populations may directly promote disease through production of pro-inflammatory cytokines, which seems to be important in the pathogenesis of PTL. T-bet, T-box transcription factor 21; Eomes, eomesodermin; CRTH2, Chemoattractant receptor-homologous molecule expressed on TH2 cells; GATA3, GATA binding protein 3; AHR, aryl hydrocarbon receptor; ROR, Retinoic acid–related orphan receptor; IFNγ, interferon-γ; IL, interleukin; LTi, lymphoid tissue inducer; NCR, natural cytotoxicity receptor; NK, natural killer; PTL, Preterm labor.

ILCs are a group of cells that share a common lymphoid progenitor. ILCs are characterized by the absence of recombination-activating gene (RAG)-dependent rearranged antigen receptors and the lack of myeloid and dendritic cell phenotypical markers, hence denominated as lineage negative (Lin^-^). ILC1s, ILC2s, and ILC3s are dependent on transcription factors T-bet, GATA-3, and RORγt, respectively. Moreover, natural killer (NK) cells are dependent on the transcription factors eomesodermin (Eomes) and T-bet for their development. These cells share the expression of a common γ chain, IL-7Rα (CD127), except for tonsil and intraepithelial ILC1 (19). In addition, ILC2 is characterized by the expression IL-2Rα (CD25), a receptor that is also present in CD56^bright^ NK cells but has a lower expression in ILC1 and ILC3 (19, 20). They are functionally diverse and belong to the innate component of the immune system (20). A summarized diagram of ILCs is presented in **Figure 1**.

ILCs were initially classified as NK cells in 1975 (21); afterward, in 1997, another cell type was added, named lymphoid tissue inducer (LTi) (22). Although NK cells represent cytotoxic ILCs capable of killing virus-infected or tumor cells and releasing pro-inflammatory cytokines (23), LTi are critical for the development of secondary lymphoid organs during embryogenesis (24). However, in the context of pregnancy, there are significant differences regarding NK function, which we address further on in this review.

ILCs play an essential role in tissue homeostasis, defense against infection and inflammation, and tissue repair (25). ILCs are mainly tissue-resident cells found in the mucosal surfaces (26) as well as in the decidua of pregnant women (27).

ILCs were classified based on their relative cytokine profiles, centered on effector phenotypes that mirror T helper cells. Over the years, the classification of ILCs has been the subject of great debate, mainly due to their heterogeneity. However, the nomenclature approved by the International Union of Immunological Societies (IUIS) considers five distinct groups: NK cells known to produce IFN-γ; group 1 (ILC1), also known to produce IFN-γ, a Th1-like cytokine; group 2 (ILC2), which are characterized by the expression of transcription factor Gata3 and the ability to produce Th2-like cytokines; group 3 (ILC3), known to produce IL-22 and IL-17; and LTis, important in secondary lymphoid organ formation (19, 25).

Moreover, it has become evident that ILCs have great plasticity. Their effector characteristics are highly dependent on their microenvironment, mainly on the cytokines secreted by tissue-resident cells, and other cells from the innate immune system (28). Due to the ability of some ILCs to produce pro-inflammatory cytokines and to the fact that ILCs express MHCII molecules, their importance in the regulation of labor is rational.

Immune tolerance and controlled inflammation are key processes in a successful pregnancy. Dysregulated inflammatory reactions often lead to complications, such as spontaneous abortion, preterm labor (PTL), preeclampsia, and intrauterine growth restriction (10, 29).

### NK Cells and ILC1

The importance of NK cells in pregnancy is paramount, not only because these cells belong to the innate immune system, but also because NK cells play an important role in placentation, remodeling of the spinal arteries, and control of trophoblast invasion (30–33). Decidual NK (dNK) cells differ substantially from peripheral NK cells: peripheral NK cells are predominantly CD56^dim^ CD16^+^, and dNK cells are CD56^bright^ CD16^-^ (23, 34). This phenotype is accompanied by functional differences because CD56^dim^ CD16^+^ cells have a strong cytolytic activity, and dNK cells are predominately cytokine-producing cells.

One key feature of dNK cells is their inability to lyse trophoblastic cells despite the expression of activating receptors (NKp46, NKp30, NKG2D, and DNAM-1) as well as their perforin and granzyme content. Instead, they produce IL-8, stromal cell–derived factor 1 (SDF-1), vascular endothelial growth factor (VEGF), and interferon gamma-induced protein 10 (IP-10), all with important roles in tissue remodeling (30, 35–37). Even though NK cells were discovered many years ago, it is only more recently that these cells were included in the ILC group. Recent work by Vento-Tormo et al. proposed three main dNK subsets: dNK1, dNK2, and dNK3 cells. This classification has been further confirmed by Huhn et al. (38, 39). Also, previous work by Yudanin et al., conducted in tissues other than uterine origin, highlights the overlapping characteristics of NK cells with ILC1, a fact also reported by Huhn et al., and this raises the question, are dNK3 subsets in fact ILC1 (40)? The nature and consequent nomenclature of the different dNK subsets and ILC1 are still a matter of great dispute.

Classically, uterine ILC1 are characterized by the expression of T-bet and Eomes and produce IFN-γ. ILC1 do not express perforin and have the inability to produce Th2- and Th17-type cytokines (27, 41). ILC1 have diverse regulatory actions dependent on the cell type and on the stimuli received (42–44).

ILC1 can be further characterized by their surface markers CD56-, CD94-, CD127+, CD117-, and they have been identified in low numbers in human decidua (27), suggesting a minor role in pregnancy (45).

### Group 2 ILCs

Group 2 ILCs are phenotypically characterized by the surface markers CD56^-^, CD127^+^, and CD161^+^ and chemoattractant receptor homologous molecules expressed on Th2 cells (CRTH2) (27, 43). ILC2 are dependent on GATA binding protein 3 and transcription factor retinoid-related orphan receptor alpha (RORα) for their development (20, 46). ILC2 produce type 2 cytokines (IL-4, IL-5, and IL-13) under the control of IL-25 and IL-33, which is important in extracellular parasitic infections and allergic responses (19). The expression of CRTH2 is of great interest for labor because it is a G protein–coupled receptor for prostaglandin D2, which promotes ILC2 differentiation and type 2 pro-inflammatory responses (47). Another important feature of the ILC2 population, found in a study conducted in a mouse model, is the expression of major histocompatibility complex class II (MHCII) as well as the co-stimulatory molecules CD80 and CD86 (48). In this study conducted by Oliphant et al*.*, it is shown that ILC2 can not only perform endocytosis, but also process and present antigens (48). These characteristics allow ILC2 to present antigens to T CD4^+^ cells and induce proliferation toward a Th2 phenotype in an IL-2-dependent manner (48). These data, albeit conducted in mouse models, reinforce the notion of cross-talk between the innate and the adaptive immune systems (49–51).

### Group 3 ILCs

Group 3 ILCs are characterized by the expression of the surface marker CD117 and the transcription factor RORγt. In a mouse model, it was proven that ILC3 express MHC class II, and they are also shown to promote T cell–mediated responses (52). Two different studies suggest that ILC3 might promote neutrophil activation with pro-angiogenic abilities, contributing to the inflammatory phase needed for implantation (53, 54). ILC3 can be further divided based on the presence of the natural cytotoxic receptor (NCR) NKp44. ILC3 NCR^+^ produce IL-22, and ILC3 NCR^-^ produce IL-17 (55, 56); both subsets have been found in human decidua (27). NCR is also present in activated peripheral NK cells and dNK. In NK cells, NCRs mediate cytotoxic (57) and antitumor responses (58); however, when present in uterine NK cells, NCR receptors have an important role in placentation through the production of IL-8, VEGF, IP-10, and SDF-1 (36).

The ability of ILC3 to act as pro-inflammatory agents (through the secretion of IL-17) suggests a preponderant role in pregnancy, which both favors embryo implantation and has an antimicrobial effect. In fact, decidual ILC3 seem to be important to pregnancy maintenance through innate defenses and tissue remodeling (27). Nevertheless, the inappropriate release of pro-inflammatory cytokines during the quiescent phase of pregnancy may prompt complications, mainly the precocious activation of the normal mechanism of labor.

## ILCs in Uterine and Fetal Compartments

*Male et al.* first made the distinction between uterine NK cells and ILC subsets in humans. In this work, ILCs were first considered precursors of uterine NK cells; however, these cells showed differences in function and phenotype through the expression of RAR related orphan receptor C (RORC), Lymphotoxin α, and IL2 genes (59), which were later attributed to ILC3 and LTi subsets. Subsequent studies identified ILC1 (60), ILC2 (61), and ILC3 (60, 61) in human endometrium and decidua based on evidence that ILCs share a common lymphoid progenitor.

ILC1 can be found in the endometrium and decidua of pregnant women as early as 9–12 weeks of gestation (27), representing an important source of IFN-γ (60) and implying a relevant role in the immune response against intracellular pathogens. In addition, the expression of CD103, an adhesion molecule that promotes the communication between lymphocytes and epithelial cells, suggests an epithelial localization of ILC1 in the endometrium and decidua (27).

Xu et al. show that, in term pregnancies, ILC2 is the most abundant population in the human decidua, and it is capable of producing Th2-type cytokines, such as IL-4, IL-5, and IL-13. In this study, the authors suggest that the pro-inflammatory qualities of ILC2 might underlie the pathological process prompting PTL (45). Specifically, Xu et al. argue that ILC populations dynamically change throughout pregnancy. In fact, they also detected ILC3 in the decidua *parietalis* that are capable of producing IL-17 and IL-22, suggesting that these cells may be responsible for inflammation-driven PTL.

ILC3 were also initially described as a subset of NK cells in the human endometrium, expressing CD127, CD161, RORC, and IL-22 (59). Later, work by Vacca et al. confirmed the ILC3 phenotype and their presence in the human endometrium and decidua during pregnancy and further divided them into two subgroups: ILC3 NKp44^+^ and ILC3 NKp44^-^ (27, 60). It is shown that, similarly to Th cells, ILCs display some degree of plasticity in response to their microenvironment. Studies conducted in mouse models show that, in response to IL-12 and IL-18, ILC3 reveal an increased expression of T-bet and decreased expression of RORγt, which results in IFN-γ production and loss of their capacity to produce IL-17 and IL-22 (62, 63). These data may explain, in part, the low numbers of ILC1 found by Xu et al. in late gestation due to overlapping functions with ILC3 phenotypes.

Amniotic fluid surrounds the embryo and fetus, protecting it mechanically during development in the event of the maternal abdomen being subject to trauma. Amniotic fluid also protects the fetus from infectious agents due to its inherent antibacterial properties (64). Amniotic fluid provides the fetus with a reservoir of fluid, nutrients, and growth factors that allow normal development and growth of fetal organs (64). The main population identified in this compartment is ILC3 of fetal origin, expressing CD127, CD117, CD161, and CD56 (64). Indeed, ILC3 are abundant in the amniotic fluid until the second trimester (64), when their numbers start to decay as gestation progresses (65). In this context, the ability of ILC3 to produce IL17 suggests a role in regulating intra-amniotic infection (64).

Fetal ILCs have been identified in the liver, secondary lymphoid organs (SLOs), intestine, lungs, and cord blood (66, 67). In the liver, ILCs assume a preponderant role because it is in this organ that hematopoiesis takes place (68, 69) and where ILC precursors (ILCPs) originate (66). In their work, Lim et al. suggest that circulating ILCP can migrate to different tissues, where they differentiate according to fetal development needs and organogenesis (66). Moreover, studies from animal models suggest that the presence of LTi cells in the fetus is essential for the successful formation of SLOs, such as the spleen, mesenteric lymph nodes, and Peyer’s patches (70–73).

Previous work has demonstrated that NK, ILC1, ILC2, and ILC3 subsets can be readily identified in the human fetal intestine (55, 64, 74, 75). It is shown that intestinal ILC2 produce IL-13 (74), and ILC3 and LTi-like cells produce IL-17A and IL-22 (55).

Mjösberg et al. report the presence of ILC2 in the fetal lung (74), and Marquardt et al. have detected increased numbers of ILC3 in the second trimester when compared to the first trimester (64).

Most of the information available regarding ILCs comes from animal models. However considering the great degree of similarity between mouse and human ILC ontology (19), we attempted a reasonable extrapolation to human biology.

The ubiquity of ILCs present in the uterine and fetal compartment denotes the importance of the innate immune system in pregnancy. Not only do ILCs take part in organ formation, but they also act as key mediators in protecting the fetus against infection and pathogens. The main findings that are the object of this review are summarized in **Table 1**.

**Table 1:** Main findings in the literature regarding human ILCs in uterine and fetal compartments.

Resident ILC population	Species	Tissue	Gestation	Main Findings	Reference
ILC1/ILC3	Human	Decidua	1^st^ Trimester	Decidual ILC3 have a frequency comparable, if not higher, with that of tonsil ILC3.Results from this study indicate that NCR^+^ ILC3 and LTi-like cells present in decidua can produce pro-inflammatory cytokines including IL-8, IL-22, IL-17A, TNF, and IFN-γ.	Vacca et al (27).
ILC3	Human	PBMCs	3^rd^ Trimester	Increased IL-17 levels observed in patients with preeclampsia, gestational diabetes, and chronic diabetes are associated with ILC3.	Barnie et al (76).
ILC1/ILC2/ILC3	Mouse/Human	Endometrium/Decidua	1^st^Trimester	CD127^+^ ILC1 are absent in human endometrium or decidua. ILC2 are found deep in the uterine wall and not in human or murine decidua, nor in human endometrium. NCR^+^ ILC3 and LTi-like ILC3 are present in both human endometrium and decidua.	Doisne et al (61).
ILC3	Human	Decidua	1^st^ Trimester	NCR^+^ ILC3 are present in decidual tissue, where they produce CXCL8 and GM -CSF, suggesting that they may have a role in neutrophil recruitment and survival.NCR^+^ ILC3-derived GM-CSF induces the expression of both heparin-binding EGF-like growth factor and IL1ra in neutrophils, important in angiogenesis and trophoblast growth/invasion.	Croxatto et al. (54)
ILC3	Human	Amniotic fluid (AF)/1^st^ and 2^nd^ trimester fetal tissue	1^st^ Trimester	CD45+ cells in AF contained very low frequencies of T cells, B cells, and monocytes.Fetal CD103+ ILC3s in AF are functional and produce high levels of IL-17 and TNF. A similar subset was identified in second trimester fetal gut and lung, suggesting that CD103+ ILC3s develop in fetal tissues and subsequently egress to the AF.	Marquardt et al (64).
ILC1/ILC2/ILC3	Human	Decidua	Term and Preterm Pregnancies	The proportion of total ILCs was increased in the decidua parietalis of women with preterm labor.ILC1s were a minor subset of decidual ILCs during preterm and term gestations; ILC2s were the most abundant ILC subset in the decidua during preterm and term gestations. The proportion of ILC2s was increased in the decidua basalis of women with preterm labor. The proportion of ILC3s was increased in the decidua parietalis of women with preterm labor; during preterm labor, ILC3s had higher expression of IL-22, IL-17A, IL-13, and IFN-γ compared to ILC2s in the decidua.	Xu et al (47).
ILC2	Human	lung and gut	–	In fetal gut, ILC2 expressed IL-13 but not IL-17 or IL-22.	Mjösberg et al (74).
ILC3	Human	Amniotic fluidIntestineLung	15 to 16Weeks	ILC3 are the main ILC population in the amniotic fluid, producing high levels of IL-17 and TNF. ILC3 are abundant in fetal intestine and lung.	Marquard et al (64).
ILC1/ILC2/ILC3	Human	Umbilical cord blood, Fetal liver	14 to 20 weeks	Human ILCPs robustly generate all ILC subsets *in vitro* and *in vivo*. This study identified unipotent ILCPs that could give rise to IFN-g^+^ ILC1s, IL-13^+^ ILC2s, or IL-17A^+^ and/or IL-22^+^ ILC3s.	Lim et al (66).
ILC1/ILC2/ILC3	Human	Liver	6 to 10weeks	In this study, the authors identified that fetal liver harbored almost exclusively NKp44− ILC3s, with ILC1s, ILC2s, and NKp44+ ILC3s being detectable only at later gestational age. Also, NKp44− ILC3s in the fetal liver were different from the corresponding population in the adult since fetal ILC3s expressed NRP1.	Forkel M. et al (67).
ILC1/ILC2/ILC3	Human	Gut	16 to 22 weeks	The study applied mass cytometry to analyze ILCs in the human fetal intestine, distinguished 34 distinct clusters and identified a previously unknown intermediate innate subset that can differentiate into ILC3 and NK cells.	Li N. et al (75).

## Innate Lymphoid Cells and the Induction of Tolerance

In order to escape the maternal immune system, trophoblast cells only express human leukocyte antigen (HLA) HLA-C, the nonclassical HLA-E, HLA-F, and HLA-G molecules (77–80).

In pregnancy, one key mechanism regulating induction of tolerance is through the actions of HLA-G molecules. The HLA-G gene is located at chromosome 6 within the class I gene cluster of MHC. HLA-G belongs to the nonclassical HLA-class I (or class Ib) genes; it is expressed mainly in the fetal–maternal interface on the extravillous cytotrophoblast (81), amnion (82), and thymus (83), and its soluble form is detectable in peripheral blood (84).

HLA-G exerts its effects by modulating antigen-presenting cells (85), suppressing proliferation of CD4^+^ T lymphocytes (86, 87), and inhibiting NK cells’ actions. In fact, HLA-G inhibits NK cells’ (84) cytolytic actions, upregulates NK inhibitory receptors (88), and is essential for implantation (89).

Also, in this perspective, there is evidence that progesterone, a key immunomodulatory steroid hormone, contributes to a pregnancy protective milieu by promoting HLA-G expression (90) and regulating NK activity (91) (92).

Tolerance is widely regarded as an adaptive response. Accordingly, it is a process that involves antigen presentation, clonal expansion, and the formation of memory cells; the expression of HLA class II molecules in ILC2 and ILC3 populations suggests that these cells might also have a role in pregnancy by presenting paternal antigens to the mother’s immune system. Although ILC2 seem capable of eliciting Th proliferation, Hepworth et al. reported, in animal models, that ILC3 lack classical costimulatory molecules, such as CD40, CD80, and CD86. If this is the case, ILC3 antigen presentation may, in fact, limit T cell responses by negatively regulating CD4+ T cell responses *in vivo* through T cell anergy (49, 93).

Whether ILCs are on the forefront in establishing tolerance toward the fetus is a matter that requires further research.

## Innate Lymphoid Cells in Disease

Studies in NK cell biology corroborate the involvement of the innate immune system in preterm birth (PTB), preeclampsia, fetal growth restriction, and morbidly adherent placentation as well as spontaneous abortion (94–99). Dysregulation or expansion of pro-inflammatory ILC populations may directly promote disease through production of pro-inflammatory cytokines, namely IL-17, which are considered important in the pathogenesis of preeclampsia and PTB (76). Moreover, high levels of IL-18 and IFN-γ have been associated with preeclampsia (100), and in PTB, there is evidence for an inadequate inflammatory response (101).

Progesterone has been known to play an important role in reproductive health for the initiation and maintenance of pregnancy with good results in the prevention of spontaneous abortion and recently in PTL (102–104).

The immunosuppressive effects of progesterone have been recognized for a long time. Despite its mode of action remaining largely unknown, progesterone has been widely adopted by clinicians around the world for prevention of PTB. Our group has already demonstrated that progesterone modulates the human T regulatory cell population during pregnancy (13, 102, 105, 106). There is also evidence, conducted in a small sample of T cell clones, suggesting that progesterone favors Th2 while dampening Th1 and Th17 responses and, thus, participates in the establishment of a favorable environment for pregnancy by its effects on T cells (107). Work from Henderson et al. shows that NK cells do not express progesterone receptors (108); however, the expression of CRTH2 in ILC2 suggests that ILCs are subject to hormonal regulation. Also, work done by Gibson et al. (109) shows that uNK cells are regulated by membrane estradiol receptors (E46), highlighting the relevance of hormone regulation in NK activity during pregnancy.

## Conclusion

Human ILCs are mainly tissue resident with relevant roles in mediating infection, inflammation, and tissue repair. Namely, ILC1 are known to promote immunity to intracellular pathogens and are associated with inflammatory bowel disease (110). ILC2 support antiparasite immunity and play an important role in airway inflammation (111). ILC3 are essential in immunity against extracellular pathogens and skin inflammation (112) and mediating graft-versus-host (113). In the past 10 years, we have witnessed a growing interest in ILC biology and their role in pregnancy. Future work, focusing on endocrine and environmental factors influencing ILC phenotype, will contribute to answer unsolved questions in clinical practice.

## Author Contributions 

JM: Scientific analysis, manuscript writing and editing. Areia: Writing and supervision of scientific content PR-S: Manuscript writing and supervising all the scientific analysis. MS-R: Writing and supervision of scientific content. AM-P: Coordination of research group, supervision of scientific content. All authors contributed to the article and approved the submitted version.

## Funding

This work was financed by Centro de Investigação em Meio Ambiente, Genética e Oncobiologia (CIMAGO) of the Faculty of Medicine of Coimbra University, through Grant Number 06/14.

## Conflict of Interest

The authors declare that the research was conducted in the absence of any commercial or financial relationships that could be construed as a potential conflict of interest.
